# The Effect of Agomelatine Treatment on Diabetes-Induced Cognitive Impairments in Rats: Concomitant Alterations in the Hippocampal Neuron Numbers

**DOI:** 10.3390/ijms19082461

**Published:** 2018-08-20

**Authors:** Özgür Devrim Can, Umut İrfan Üçel, Ümide Demir Özkay, Emel Ulupınar

**Affiliations:** 1 Department of Pharmacology, Faculty of Pharmacy, Anadolu University, 26470 Eskişehir, Turkey; uiucel@anadolu.edu.tr (U.İ.Ü.); udemir@anadolu.edu.tr (Ü.D.Ö.); 2 Department of Anatomy, Faculty of Medicine, Eskisehir Osmangazi University, 26480 Eskisehir, Turkey; eulupi@ogu.edu.tr; 3 Interdisciplinary Neuroscience Department, Health Science Institute of Eskişehir Osmangazi University, 26480 Eskisehir, Turkey

**Keywords:** diabetes mellitus, hippocampus, learning, memory, optic fractionator

## Abstract

Researches that are related to the central nervous system complications of diabetes have indicated higher incidence of cognitive disorders in patients. Since the variety of nootropic drugs used in clinics is limited and none of them consistently improves the outcomes, new and effective drug alternatives are needed for the treatment of diabetes-induced cognitive disorders. Based on the nootropic potential of agomelatine, the promising efficacy of this drug on cognitive impairments of diabetic rats was investigated in the current study. Experimental diabetes model was induced by streptozotocin. After development of diabetes-related cognitive impairments in rats, agomelatine (40 and 80 mg/kg) was administrated orally for two weeks. Cognitive performance was assessed by Morris water-maze and passive avoidance tests. Then, the total numbers of neurons in both dentate gyrus and Cornu Ammonis (CA) 1–3 subfields of the hippocampus were estimated by the optical fractionator method. Agomelatine treatment induced notable enhancement in the learning and memory performance of diabetic rats. Moreover, it reversed the neuronal loss in the hippocampal subregions of diabetic animals. Obtained results suggest that agomelatine has a significant potential for the treatment of diabetes-induced cognitive impairments. However, therapeutic efficacy of this drug in diabetic patients suffering from cognitive dysfunctions needs to be confirmed by further clinical trials.

## 1. Introduction

Diabetes mellitus (DM) is a disorder in which abnormalities in carbohydrate, fat, and protein metabolisms develop due to the deficiency or dysfunction of the insulin hormone [1]. Having a high prevalence in the worldwide, DM has a special clinical importance in terms of the complications it causes. Acute complications of DM, which are caused by absolute or relative deficiency of insulin, are diabetic ketoacidosis, hyperosmolar non-ketotic syndrome, and comas induced by lactic acidosis or hypoglycaemia [2]. DM can also lead to chronic complications that are characterized by impaired and insufficient functions of the various systems, organs, and tissues due to the long-term metabolic disturbances. Cardiovascular, cerebrovascular, and peripheral vascular diseases as well as retinopathy, neuropathy, and nephropathy can be listed as the major chronic complications of DM [2]. Today, harmful effects of DM on the central nervous system (CNS), entitled as diabetic encephalopathy [3], is also classified as a chronic complication of DM [4]. 

Detrimental effect of DM on the mood and mental health of patients has been widely investigated, so far. Currently, affective disorders, such as depression and anxiety, are considered as some of the most common comorbidities of diabetes [5,6,7]. In addition to unfavorable effects on the emotional state, DM affects the cognitive performances of the patients in a negative way. It has been shown that attenuation in the learning capacities and memory consolidation processing, as well as impairments in problem solving occurs in patients with Type I DM [8]. Similar to Type I, patients with Type II DM also suffer from the weakening of cognitive functions, insufficiency in abstract judgments, and complex psychomotor activity, and finally greater risk of dementia. It has been reported that impairments in the complex cognitive tasks requiring data storage and creating new information are especially declined in these patients [9,10,11]. Although, diabetes-induced deteriorations in the mental health of patients are well-described by several previous studies, no specific nootropic drug or a treatment protocol is present to improve the outcomes. Therefore, developing new agents or repositioning of the currently used drugs for the treatment of diabetes-induced cognitive disorders has an increasing interest. 

Agomelatine, a structural analog of melatonin, is a relatively new drug that is prescribed for the management of depressive disorders. This drug also has noteworthy therapeutic efficacy for the treatment of generalized anxiety disorder [12]. It has a unique pharmacological mechanism by having agonistic effects on melatonergic MT_1_ and MT_2_ receptors, but antagonistic effects on serotonergic 5-HT_2C_ receptors [13,14]. As well as notable therapeutic efficacy of agomelatine on emotional disorders, chronic administration of this drug has been shown to induce notable neuroprotection, improve learning, activate molecular mechanisms of memory storage, and facilitate episodic memory [15,16,17]. Our research group has also shown that chronic agomelatine administration enhances cognitive performance and hippocampal plasticity by increasing the density of mushroom and stubby types of spines in the pyramidal neurons [18]. Furthermore, various animal models, including glucocorticoid receptor-impairment [19], prenatal stress [20], and corticosterone treatment induced depression/anxiety [21], have confirmed the nootropic efficacy of agomelatine. However, little is known about its potential on diabetes-related cognitive dysfunctions. Therefore, we aimed to investigate the promising therapeutic activity of agomelatine on cognitive impairments induced by diabetes. In addition to behavioral tests, total number of neurons in the hippocampus, one of the key areas for learning and memory formation in the brain, were examined by using unbiased stereological quantification methods.

## 2. Results and Discussion

In this study, the effect of agomelatine treatment on chronic hyperglycemia-induced neurobehavioral alterations was investigated in an experimental model of diabetes, which was established by streptozotocin (STZ, a N-nitroso derivative of glucosamine). This toxin is known to cause selective damage to pancreatic β cells, and therefore, induce hypoinsulinemia and hyperglycemia in rodents [22,23]. STZ-induced diabetes is an extensively used experimental model in neurochemical, electrophysiological, morphological, and behavioral studies [24,25,26,27]. Since STZ rapidly eliminated from the body and cannot cross the blood–brain barrier, the differences seen in diabetic animals are assumed to originate from diabetes itself, not from STZ [28].

Piracetam, which is the most common of the nootropic drugs [29,30], is known to modulate a range of neurotransmission systems and has neuroprotective and neuroplastic properties [31]. Since it is widely preferred reference drug in several animal models of cognitive disorder [30,32,33], we chose piracetam (a cyclic derivative of γ-aminobutyric acid, GABA), as a positive control, to compare the effects of agomelatine in this study.

**Figure 1:**
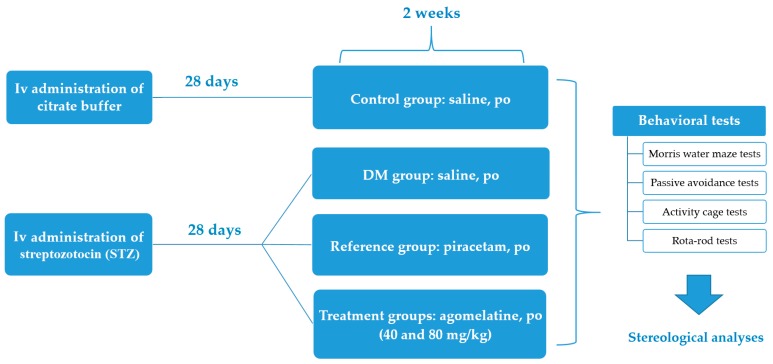
Brief summary of the experimental design.

Details of the experimental settings and treatment protocols were presented in Figure 1.

### 2.1. The Improving Effect of Agomelatine Treatment on Learning and Memory Impairements in Diabetic Rats

**Figure 2:**
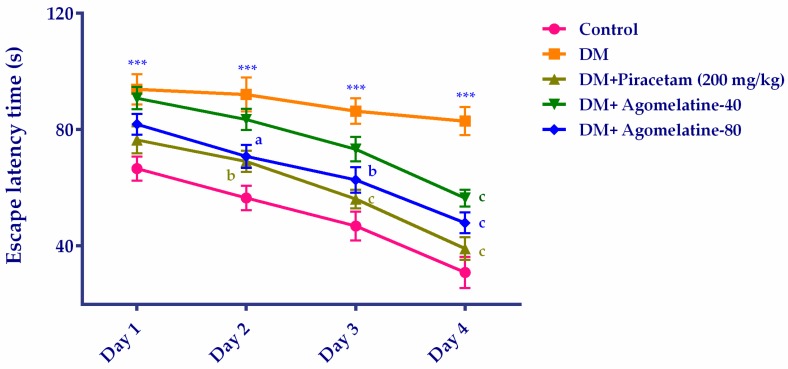
Escape latency time of normoglycemic rats administered saline solution (control) and diabetic rats administered saline (DM), 200 mg/kg piracetam, 40 mg/kg agomelatine or 80 mg/kg agomelatine for 2 weeks, in the Morris Water Maze (MWM) test. Values are given as mean ± S.E.M. Significant difference against corresponding control group *** *p* < 0.001; Significant difference against corresponding DM group ^a^ *p* < 0.05, ^b^ *p* < 0.01, ^c^ *p* < 0.001. Two-way repeated-measures ANOVA, post-hoc Bonferroni multiple comparison test, *n* = 8.

Spatial learning and memory performances of animals were evaluated by Morris Water Maze (MWM) test, one of the most extensively used methods in behavioral studies [34]. In the MWM tests, “escape latency time” of experimental groups were compared by two-way repeated ANOVA (Figure 2). Results of the analysis revealed that both treatment [F (12,105) = 64.52, *p* < 0.001] and time [F (12,105) = 43.67, *p* < 0.001] factors affected the escape latency time of rats. No significant interaction was observed between these factors [F (12,105) = 1.136, *p* > 0.05].

Multiple comparisons of groups by Bonferroni tests showed that the escape latency of untreated diabetic rats was significantly higher than that of normoglycemic control animals in all of the test days. On the other hand, diabetic rats receiving agomelatine at 80 mg/kg dose or reference drug piracetam for two weeks, have found the hidden platform faster than the untreated diabetic animals on the 2nd, 3rd, and 4th days of the MWM tests. In diabetic rats receiving agomelatine at 40 mg/kg doses, escape latency was significantly reduced only on the 4th day of the tests (Figure 2).

**Figure 3:**
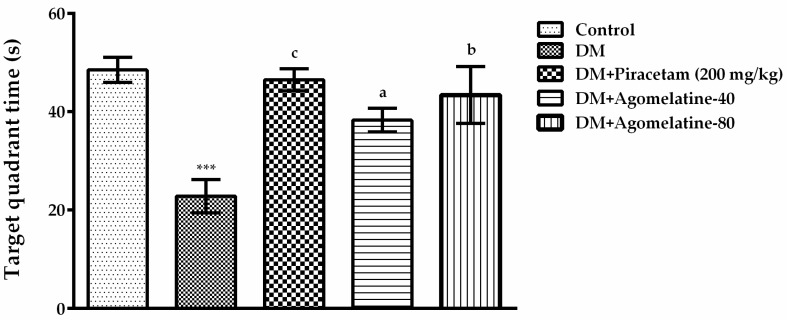
Target quadrant time of normoglycemic rats administered saline solution (control) and diabetic rats administered saline (DM), 200 mg/kg piracetam, 40 mg/kg agomelatine or 80 mg/kg agomelatine for two weeks, in the MWM test. Values are given as mean ± S.E.M. Significant difference against control group *** *p* < 0.001; Significant difference against DM group ^a^ *p* < 0.05, ^b^ *p* < 0.01, ^c^ *p* < 0.001. One-way-ANOVA, post-hoc Tukey HSD multiple comparison test, *n* = 8.

“Target quadrant time” of the experimental groups in MWM tests was compared by one-way ANOVA [F (4,39) = 15.16, *p* < 0.001]. The results of the Tukey HSD multiple comparison tests exhibited that diabetic rats spent significantly less time in the target quadrant than those of normoglycemic controls (*p* < 0.001). On the other hand, administration of agomelatine at 40 mg/kg (*p* < 0.05) or 80 mg/kg (*p* < 0.01) doses induced a significant increase in the target quadrant time of diabetic rats. Diabetic rats receiving piracetam spent significantly (*p* < 0.001) longer time in the target quadrant, as expected (Figure 3).

Our MWM test results indicated a deterioration in the learning and memory performance of diabetic rats in compatible with the previous reports in the literature [35,36,37]. Besides, agomelatine treatment effectively reversed the impaired learning and memory performance of diabetic rats, similar to reference drug piracetam.

**Figure 4:**
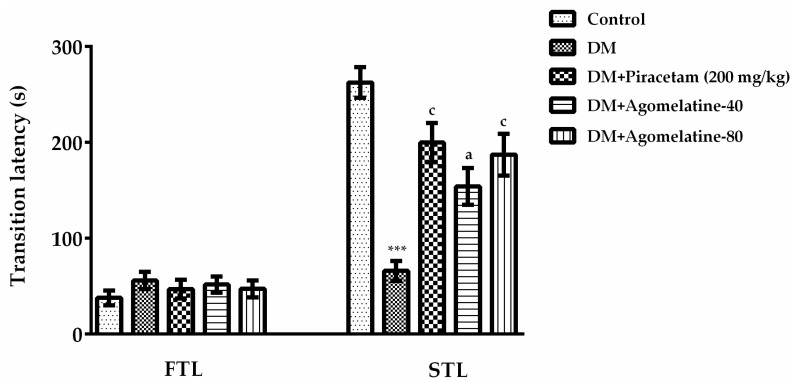
First transition latency (FTL) and second transition latency (STL) values of normoglycemic rats administered saline solution (control) and diabetic rats administered saline (DM), 200 mg/kg piracetam, 40 mg/kg agomelatine or 80 mg/kg agomelatine for two weeks, in the passive avoidance test. Values are given as mean ± S.E.M. Significant difference against control group *** *p* < 0.001; Significant difference against DM group ^a^ *p* < 0.05, ^c^ *p* < 0.001. One-way-ANOVA, post-hoc Tukey HSD multiple comparison test, *n* = 8.

In the second step of our study, the cognitive performances of diabetic rats were assessed further, by using the passive avoidance method. The passive avoidance task is a fear-aggravated test that is widely used to evaluate emotional learning and memory functions in experimental animals [34]. In the passive avoidance tests, “transition latency” values of the experimental groups were compared by one-way ANOVA [F (4,39) = 16.02, *p* < 0.001] (Figure 4). Results of the Tukey HSD multiple comparison tests exhibited that there is no difference in the “first transition latency” values of the animals between the experimental groups. Whereas, second transition latencies of diabetic rats into the dark compartment were significantly shorter than those of the normoglycemic controls (*p* < 0.001). Treating the diabetic rats with 40 mg/kg (*p* < 0.05) and 80 mg/kg (*p* < 0.001) doses of agomelatine reversed this reduction and prolonged the entrance latency to the dark compartment. Second transition latency of diabetic rats treated with piracetam is also longer than those of untreated diabetic rats (*p* < 0.001). These findings indicated that diabetes caused impairments in the emotional learning and memory performances of rats in parallel with the previous literature [38,39]. In addition, agomelatine treatment reversed these detrimental effects with a comparable efficacy to piracetam.

### 2.2. Unchanged Motor Activity in Diabetic Rats Following Agomelatine Treatment

It is known that possible changes in the motor activities of the experimental animals can affect the test results that were performed to evaluate cognitive performance [40]. For this reason, monitoring the motor activity of rodents during behavioral experiments has a great importance. Therefore, we assessed both spontaneous locomotor activities and motor coordination of rats that were placed in our experimental groups.

**Figure 5:**
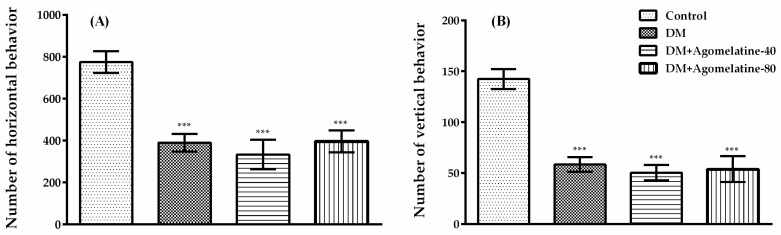
Number of (**A**) horizontal and (**B**) vertical locomotor activities of normoglycemic rats administered saline solution (control) and diabetic rats administered saline (DM), 40 mg/kg agomelatine or 80 mg/kg agomelatine for two weeks, in the activity cage test. Values are given as mean ± S.E.M. Significant difference against control group *** *p* < 0.001. One-way-ANOVA, post-hoc Tukey HSD multiple comparison test, *n* = 8.

Horizontal (Figure 5A) and vertical (Figure 5B) spontaneous locomotor activity counts of the rats were compared with one-way-ANOVA ([F (3,31) = 13.46, *p* < 0.001] and [F (3,31) = 21.43, *p* < 0.001]). The results of the Tukey HSD multiple comparison test indicated that both of the horizontal (*p* < 0.001) and vertical (*p* < 0.001) spontaneous locomotor activity numbers of diabetic rats significantly decreased compared to those of normoglycemic control animals. Administration of agomelatine for two weeks, neither at 40 mg/kg nor at 80 mg/kg doses, caused an additional change in the locomotor activities of these animals.

**Figure 6:**
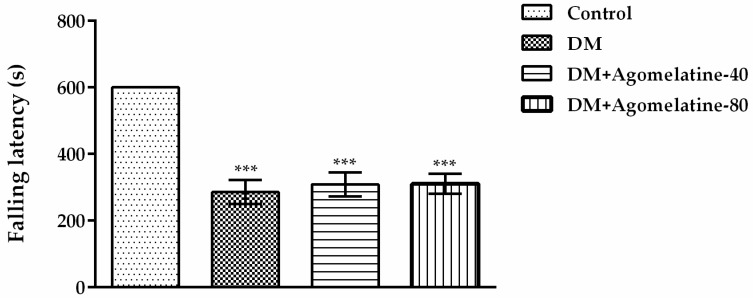
Falling latencies of normoglycemic rats administered saline solution (control) and diabetic rats administered saline (DM), 40 mg/kg agomelatine or 80 mg/kg agomelatine for 2 weeks, in the Rota-rod test. Values are given as mean ± S.E.M. Significant difference against control group *** *p* < 0.001. One-way-ANOVA, post-hoc Tukey HSD multiple comparison test, *n* = 8.

Motor coordination parameters of rats (Figure 6) were analyzed by one-way-ANOVA [F (3,31) = 25.42, *p* < 0.001]. Subsequent Tukey HSD multiple comparisons displayed a significant reduction in falling latency values of diabetic rats compared to those of normoglycemic animals (*p* < 0.001). Nevertheless, the administration of agomelatine for two weeks did not cause any additional changes.

Obtained data from activity cage and Rota-rod tests supported the results of the previous studies reporting impaired motor activity and motor coordination in diabetic experimental animals [41,42,43]. On the other hand, administration of agomelatine caused no significant change either in the total number of locomotor activities or in the falling latencies of diabetic animals, indicating that the nootropic effect of this drug was not affected by motor activity performances of the animals.

In summary, the results of the behavioral experiments suggested that agomelatine successfully retrieves the diabetes-induced impairments in the spatial and emotional learning and memory performance of rats without influencing their motor activity.

After completing the behavioral tests, we have conducted further stereologic evaluations in the hippocampal subregions to examine the effect of agomelatine treatment at cellular level.

### 2.3. Recovery of the Hippocampal Neuronal Loss Following Agomelatine Treatment in Diabetic Rats

**Figure 7:**
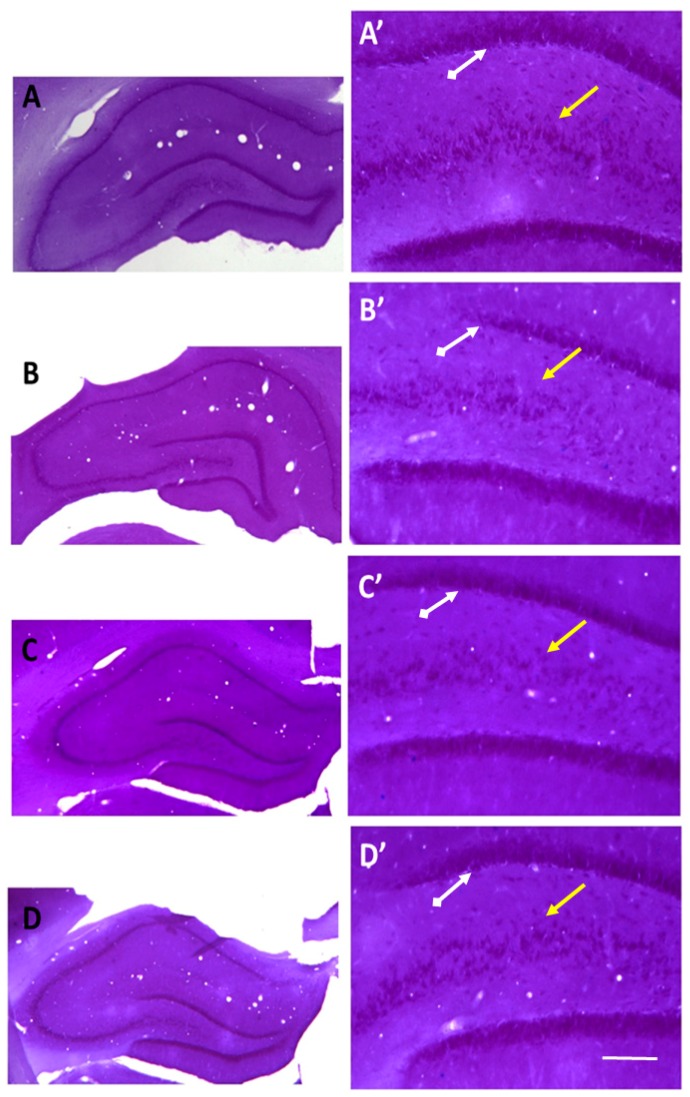
Photomicroscopic view of the hippocampus in the normoglycemic rats administered saline solution (**A**) and diabetic rats administered saline (**B**), 40 mg/kg agomelatine (**C**) or 80 mg/kg agomelatine (**D**). Scale bar = 80 microns in (**A**–**D**) and 20 microns in **A’**–**D’**. White arrows display the CA1–3 subregions of the hippocampus while yellow arrows indicate the dentate gyrus. In comparison to diabetic rats, in control and agomelatine-treated groups pyramidal cells and granule cells were more densely packed in CA1–3 and dentate gyrus, respectively.

Histological alterations in the hippocampal subfields were examined in Nissl-stained sections and light microscopic views of representative sections were shown in Figure 7. Structural integrity of the hippocampus appeared comparable in normoglycemic (Figure 7A), diabetic (Figure 7B), and agomelatine-treated diabetic animals (Figure 7C,D). On the other hand, high-power view of the dentate gyrus and CA1–3 subfields (enlarged boxes, Figure 7A’–D’) showed more densely packed cells and a more intense staining pattern in the normoglycemic and agomelatine-treated diabetic animals when compared to the untreated ones. To further confirm these qualitative observations, we used modern design-based stereological methods and estimated the total number of cells in the CA1-3 and dentate gyrus regions, respectively.

**Figure 8:**
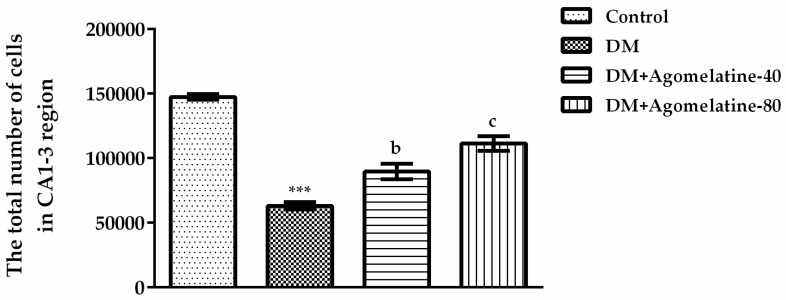
The total number of cells in the CA1–3 region of normoglycemic rats administered saline solution (control) and diabetic rats administered saline (DM), 40 mg/kg agomelatine or 80 mg/kg agomelatine for 2 weeks. Values are given as mean ± S.E.M. Significant difference against control group *** *p* < 0.001. Significant difference against DM group ^b^ *p* < 0.01; ^c^ *p* < 0.001. One-way-ANOVA, post-hoc Tukey HSD multiple comparison test, *n* = 6.

Differences of the total cell numbers in the CA1–3 regions were displayed in Figure 8 [F (3,23) = 64.27, *p* < 0.001]. The results of the Tukey HSD multiple comparison test revealed that, in diabetic animals, the total number of cells in the CA1–3 regions was significantly reduced relative to those of normoglycemic control animals (*p* < 0.001). However, the administration of 40 mg/kg (*p* < 0.01) and 80 mg/kg (*p* < 0.001) agomelatine for two weeks reversed this diabetes-induced reduction (Figure 8).

**Figure 9:**
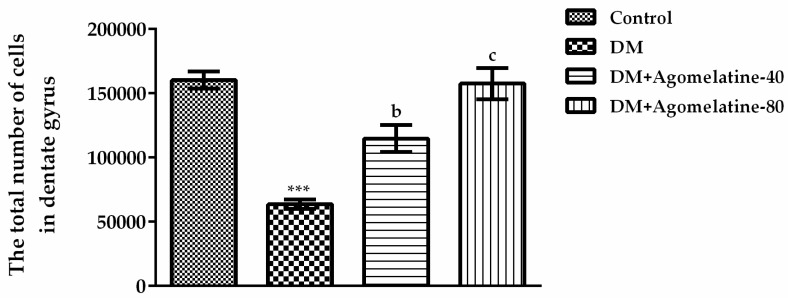
The total number of cells in the dentate gyrus of normoglycemic rats administered saline solution (control) and diabetic rats administered saline (DM), 40 mg/kg agomelatine or 80 mg/kg agomelatine for 2 weeks. Values are given as mean ± S.E.M. Significant difference against control group *** *p* < 0.001. Significant difference against DM group ^b^ *p* < 0.01; ^c^ *p* < 0.001. One-way-ANOVA, post-hoc Tukey HSD multiple comparison test, *n* = 6.

Figure 9 exhibited the comparisons of the total cell numbers in the dentate gyrus [F (3,23) = 26.13, *p* < 0.001]. The results of the Tukey HSD multiple comparison test showed that the total number of cells at hippocampal dentate gyrus in diabetic animals was decreased relative to those of normoglycemic control animals (*p* < 0.001). Administration of 40 mg/kg (*p* < 0.01) and 80 mg/kg (*p* < 0.001) agomelatine for two weeks significantly reversed this diabetes-induced decrease in the dentate gyrus (Figure 9).

Obtained stereological findings are in consistent with the previous reports displaying diabetes-induced apoptosis and severe neuronal losses in the hippocampus of rodents [44,45,46]. These negative effects of diabetes on hippocampus seem related to the cognitive deficits of rats. On the other hand, agomelatine treatment successfully reversed the neuronal loss in the hippocampal subregions. Although optical fractionator results implies an enhancement in the survival rate of diabetic neurons following agomelatine treatment, the exact mechanisms underlying this effect is not known. It should be noted that diabetic neurodegeneration could create massive neuroinflammation and the consequent activation of glial and/or microglial cells might cause a neuron-threatening environment [47,48]. Melatonin has been shown to possess beneficial effects on diabetes-related changes in the amount and composition of specific neural and glial proteins playing role in the development of cognitive deficits [49]. Therefore, it is possible that agomelatine, which is an analog of melatonin, might diminish the neuro-inflammatory processes in the hippocampus. This possibility needs to be clarified with further immune-histochemical labelling studies using well-established markers for inflammatory cells, such as glial fibrillary acidic protein (GFAP), Iba1 (ionized calcium binding adaptor molecule 1), and CD45. 

Earlier studies have demonstrated the dramatic reductions in the neurogenesis and cell proliferation in the dentate gyrus of diabetic rodents [44,45,46]. On the other hand, it has been reported that agomelatine has a notable capacity to enhance neurogenesis in the hippocampus [18,19,20,50,51,52]. Melatonin agonist properties of this drug have been reported to mediate new granule cell maturation and survival, possibly through the upregulation of brain-derived neurotrophic factor (BDNF) levels [53]. Besides, 5-HT_2C_ antagonistic actions of this drug has been predominantly associated with the cell proliferation in the hippocampus [51,54]. Based on these dual effects on melatonergic and serotonergic receptors, our quantitative results in the total number of hippocampal neurons might reflects both survival and proliferation inducing capacity of agomelatine. However, cell type specific proliferative activity of agomelatine within the subgranuler zone of dentate gyrus should be clarified by using specific neurogenesis markers, such as Ki67, 5-Bromo-2’-deoxyuridine (BrdU), GFAP, Sox1 (a marker for neural stem cells), polysialylated neural cell adhesion molecule (PSA-NCAM), or doublecortin (DCX), in future studies.

It is a general knowledge that dynamic changes in synaptic strength play a vital role in learning and memory processes. However, diabetes induces severe damage to hippocampal synaptic plasticity via various pathological mechanisms [35,55,56]. Recently, our research group has shown that chronic administration of agomelatine increases the mushroom and stubby spine densities of hippocampal pyramidal neuron dendrites, indicating a consequent augmentation in the synaptic plasticity [18]. These results are in accordance with the findings of some other laboratories. For example, Martin and co-workers have demonstrated that agomelatine improves the stress-induced cognitive dysfunction in mice, probably through a mechanism involving BDNF signaling, synaptic plasticity, and epigenetic remodeling [57]. Furthermore, this drug has been shown to normalize stress related changes in the hippocampal neuroplasticity-related genes, such as activity-regulated cytoskeleton-associated protein (Arc), B-cell lymphoma 2 (Bcl2), BDNF, glial cell line-derived neurotrophic factor (GDNF), insulin-like growth factor 1 (IGF1), and neurogenic differentiation factor 1 (NEUROD1) [58]. It has also been reversed the stress-induced decrease in synapsin-1 levels, which is a regulator of synaptic transmission and plasticity, in the hippocampal dentate gyrus [59,60]. Favorable effect of agomelatine on neuroplasticity has also been shown in the prefrontal cortex, as well as the hippocampus [61]. Therefore, the beneficial effect of agomelatine on diabetes-induced cognitive dysfunction might probably be related to the hippocampal plastic changes. This assumption also has to be confirmed by additional studies.

In summary, findings of the present study demonstrated, for the first time, that agomelatine has a noteworthy therapeutic potential on diabetes-induced cognitive impairments and hippocampal neuronal loss. Augmented levels of neurotrophic factors supporting neuronal survival such as BDNF, fibroblast growth factor 2 (FGF-2) or neural cell adhesion molecule (NCAM) [15,19,50,51,62], strengthening synaptic plasticity, and enhancing neurogenesis in the hippocampus [18,19,20,50,51,52] induced by agomelatine administration might mediate the nootropic efficacy of this drug against diabetes-induced cognitive decline. However, exact mechanisms of action need to be clarified with further detailed studies.

## 3. Materials and Methods

### 3.1. Animals

Male Sprague-Dawley rats weighing 250–300 g were used in this study. The animals were housed in a temperature controlled (24 ± 1 °C), well-ventilated rooms in 12 h light/dark cycle (lights switched on between 8^00^–20^00^). They had *ad libitum* access to food and water, except in the course of the experimental sessions. The experimental protocol of this study was evaluated and approved by the Anadolu University Animal Experiments Local Ethics Committee (Date: 8 December 2014 and Project identification code: 2014-40).

### 3.2. Chemicals

In this study, STZ, piracetam, halothane, paraformaldehyde (Sigma-Aldrich, St. Louis, MO, USA), citric acid, trisodium citrate, ethanol, xylene, crystal violet (Merck, Darmstadt, Germany), and physiological saline solution (Adeka, Samsun, Turkey) were used. Agomelatine was purchased from Servier Company (Valdoxan^®^, Wexham, Slough, UK).

### 3.3. Induction of Experimental Diabetes Model in Rats

Experimental diabetes model was induced in rats, as described previously [63]. A single dose of 50 mg/kg STZ, prepared in pH = 4.5, 0.1 M citrate buffer, was injected into the tail vein of the rats. After this injection, water bottles containing a solution of 5 mmol/L glucose were placed to the cages with the aim of preventing hypoglycemia. Blood samples were taken from the tail of the rats 72 h after the STZ administrations and glucose levels were measured by Glukotrend^®^ (Roche, Basel, Switzerland). Animals with a blood glucose level above 300 mg/dL were regarded as diabetic. The same volume of citrate buffer was injected (i.v.) to the control group, since STZ was dissolved in it.

### 3.4. Experimental Groups

Experimental groups were designed as control group, DM group, reference drug piracetam (200 mg/kg, i.p.) treated group (DM + Piracetam) [30], 40 mg/kg p.o. agomelatine treated group (DM + Agomelatine-40), and 80 mg/kg p.o. agomelatine treated group (DM + Agomelatine-80) [64].

### 3.5. Behavioral Tests

#### 3.5.1. Morris Water Maze (MWM) Test

MWM method is used to assess the spatial learning and memory functions of the experimental animals. The device (Ugo-basile, 40155, Verase, Italy) consists of a circular tank with a height of 60 cm and a diameter of 150 cm. Throughout the tests, the tank was filled up with 25 ± 1 °C water. A cylindrical escape platform was placed 2 cm below the water level and the water in the tank was opacified with milk dust to prevent the platform from appearing.

MWM test was conducted, as described previously [65]. Briefly, during the first four days of the test protocol, rats were subjected to four consecutive trials at five minute intervals every day. For these trials, tank is divided into four equal quadrants hypothetically and animals were gently placed into the water, facing toward the wall of the pool, from one of the quadrants. Every new trial was started in the following quadrant. The rats were observed for maximum of 120 s to find the escape platform. The animal that find the hidden platform was allowed to remain on it for 20 s. Time elapsed to find the hidden platform was recorded as “escape latency”. If the animals can not discover within 120 s, they were manually directed to the platform and were then permitted to stay on it for 20 s. Following the four-day acquisition experiments, on the 5th day, the platform was taken out of the tank and animals were allowed to swim freely in the tank for 120 s. Time spent in the target quadrant was recorded as “target quadrant time”.

#### 3.5.2. Passive Avoidance Test

Passive avoidance test was used to assess fear-conditioned emotional memory [66]. The apparatus (Ugo Basile, 7550, Verase, Italy) consists of two different compartments (22 cm × 21 cm × 22 cm): an illuminated white compartment and a dark compartment. These compartments are separated by a flat section carrying an automatically operated sliding door at the floor. In the dark compartment, which has a grill floor, an unavoidable electric current (0.5 mA) can be applied to feet of the animals via a shock generator.

Experimental session was started with the training trials. On the first day of training, rats were placed in the light compartment for 30 s, and then the door between the compartments was opened for allowing animals to move freely into the dark area. 15 min after this first trial, the animal was placed in the light compartment once more for the acquisition experiment. After a 30 s acclimation period, the door was opened and the time spent for passing to the dark compartment was recorded as “first transition latency”. If this entrance did not occur within 300 s, the animal was eliminated from the experiment. When the animal completely entered to the dark compartment, the door was automatically closed and an electric foot shock (0.5 mA) was applied to the animal’s feet through the grid floor for 3 s.

A retention trial was conducted 24 h after the acquisition trial. This time, the rat was placed in the light compartment and entry time to the dark compartment was recorded as “second transition latency”. The rats were given a maximum of 300 s to enter the dark compartment. Among the trials, each of the compartments was cleaned to remove possible olfactory cues.

#### 3.5.3. Activity Cage Test

Spontaneous locomotor activities of the animals in vertical and horizontal directions were recorded for 10 min by an activity cage apparatus (Ugo Basile, 7420, Verase, Italy) [63].

#### 3.5.4. Rota-rod Test

Motor coordination of rats were evaluated by a Rota-rod device (Ugo Basile, 47750, Verase, Italy), setting at a constant rate of 8 rpm. First, animals were trained for staying over a rotating mill, for three consecutive days. Then, latency to fall from the mill was recorded as a parameter for motor coordination [67].

### 3.6. Morphological Methods

#### 3.6.1. Tissue Processing and Nissl Staining

After the behavioral experiments, the rats were anesthetized with halothane. Following the transcardial perfusion with 0.1 M phosphate buffer (pH 7.4), they were fixed with 4% paraformaldehyde in phosphate buffer. Brains were dissected, fixed overnight in 4% paraformaldehyde, and then cryoprotected in phosphate buffer containing 30% sucrose.

Coronal sections of the hippocampus were cut sequentially at the thickness of 100 μm with the aid of a Vibratome (Pelco 10190). Using the systematic random sampling method, every 4th section (with 400-μm intervals) was mounted on poly-l-lysine-coated slides. The slides were allowed to air dry at room temperature, then serially rehydrated in 100%, 95% and 50% alcohol/distilled water and stained with 0.1% cresyl violet solution for 5 min. Samples were rinsed with distilled water and then allowed to differentiate in 70% ethyl alcohol containing several drops of glacial acetic acid for 2–5 min. Afterwards, samples were dehydrated in 70%, 95% and 100% ethanol, cleaned with xylene, and covered with permanent mounting medium [65].

#### 3.6.2. Optical Fractionator Counting Method

**Figure 10:**
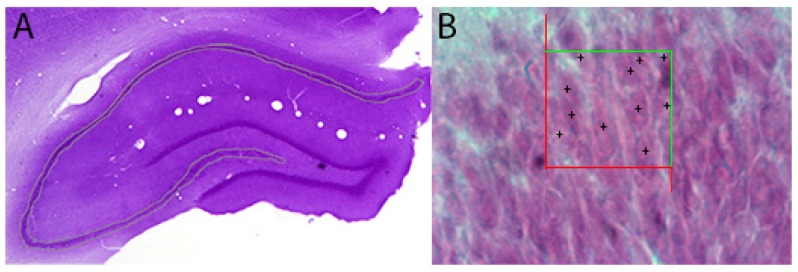
Total number of cells in the CA1–3 and dentate gyrus subfields of the hippocampus is estimated by the optical fractionator method. Hippocampal regions were outlined by 2.5× objective (**A**) and then number of particles in the counting frame (30 × 30 μm) was counted. Representative image displays the counting frame in the dentate gyrus and inclusion or exclusion of granular cells in the acceptance (green) and rejection (red) lines was shown in (**B**).

Neurons of the hippocampal formation was counted along the left hippocampus axis by using the optical fractionator probe of the Stereo Investigator system (MBF Bioscience). First, the contours of the dentate gyrus and the CA1–3 subfields were delineated under low magnification (2.5×) (Figure 10A). Then, approximately 60% of the outlined region was analyzed by systematic random sampling [38]. All neuronal profiles in every 8th sections were counted while using an oil-immersion lens (Figure 10B) [68].

The total number of cells in the pyramidal (CA1–3) and granular cell layers (dentate gyrus) in the left hemisphere was calculated using the following formula with the Stereo Investigator program:

*N* = (1/ssf). (1/asf). (1/hsf). EQ-.

*N*: number of neurons; ssf: the section sampling fraction; asf: the area sampling fraction, which was calculated by dividing the area sampled by the total area of the respective subfield, which is a (frame)/a (x,y step); hsf: the height sampling fraction calculated by dividing the height (30 µm in this study) by the section thickness at the point of analysis; and, EQ-: the total count of particles sampled for the entire subfield.

The Coefficient of Error (CE) was calculated according to Schmitz and Hof [69] and values less than 0.10 were considered to be acceptable.

### 3.7. Statistical Analysis

GraphPad Prism 6.01 (GraphPad Software, San Diego, CA, USA) program was used for statistical analyses and graphic drawings. The escape latency values of rats measured in the MWM were analyzed using two-way repeated-measures analysis of variance, followed by the Bonferroni post-hoc test. The rest of the experimental data were evaluated by one-way ANOVA, followed by the Tukey HSD multiple comparison test. Results are given as the mean ± standard error of mean (S.E.M.). A value of *p* < 0.05 was considered as significant.

## 4. Conclusions

Results of this study indicate that agomelatine, comparable to piracetam, has a potential for the treatment of diabetic patients suffering from cognitive dysfunctions. Recently, we have reported that agomelatine may enhance the medical comfort of diabetic patients via its antihyperalgesic and antiallodynic activities against painful diabetic neuropathy [63]. Therefore, the capacity of agomelatine in the treatment of both neuropathic pain and cognitive dysfunctions can make this drug as an ideal alternative for diabetic patients. Moreover, empirical findings demonstrating the ineffectiveness of agomelatine on the blood glucose levels of healthy or diabetic animals [63] are also important in terms of indicating that the use of this drug may not cause a disruptive effect on glycemic control of patients with diabetes. Therefore, following well-designed clinical trials, agomelatine might be considered as a favored drug for the treatment of diabetic patients due to its specific therapeutic advantages.

## Abbreviations

ANOVA	Analysis of variance
Arc	Activity-regulated cytoskeleton-associated protein
Bcl2	B-cell lymphoma 2
BDNF	Brain-derived neurotrophic factor
BrdU	5-Bromo-2’-deoxyuridine
CA	Cornu Ammonis
CD45	Lymphocyte common antigen
CNS	Central nervous system
DCX	Doublecortin
DM	Diabetes mellitus
FGF-2	Fibroblast growth factor 2
FTL	First transition latency
GABA	γ-aminobutyric acid
GDNF	Glial cell line-derived neurotrophic factor
GFAP	Glial fibrillary acidic protein
5-HT_2C_	Serotonergic receptor subtype
Iba1	Ionized calcium binding adaptor molecule 1
IGF1	Insulin-like growth factor 1
MT_1_, MT_2_	Melatonergic receptor subtypes
MWM	Morris Water Maze
NCAM	Neural cell adhesion molecule
NEUROD1	Neurogenic differentiation factor 1
PSA-NCAM	Polysialylated neural cell adhesion molecule
S.E.M.	Standard error of mean
STL	Second transition latency
STZ	Streptozotocin
